# Pending Reorganization of *Hantaviridae* to Include Only Completely Sequenced Viruses: A Call to Action

**DOI:** 10.3390/v15030660

**Published:** 2023-02-28

**Authors:** Jens H. Kuhn, Steven B. Bradfute, Charles H. Calisher, Boris Klempa, Jonas Klingström, Lies Laenen, Gustavo Palacios, Connie S. Schmaljohn, Nicole D. Tischler, Piet Maes

**Affiliations:** 1 Integrated Research Facility at Fort Detrick, Division of Clinical Research, National Institute of Allergy and Infectious Diseases, National Institutes of Health, Fort Detrick, Frederick, MD 21702, USA; 2 Department of Internal Medicine, University of New Mexico Health Sciences Center, Albuquerque, NM 87131, USA; 3 Colorado State University, Fort Collins, CO 80523, USA; 4 Institute of Virology, Biomedical Research Center, Slovak Academy of Sciences, 84505 Bratislava, Slovakia; 5 Division of Molecular Medicine and Virology, Department of Biomedical and Clinical Sciences, Linköping University, 581 83 Linköping, Sweden; 6 Zoonotic Infectious Diseases Unit, KU Leuven, Rega Institute, 3000 Leuven, Belgium; 7 Belgium Department of Laboratory Medicine, University Hospitals Leuven, 3000 Leuven, Belgium; 8 Department of Microbiology, Icahn School of Medicine at Mount Sinai, New York, NY 10029, USA; 9 Global Health Emerging Pathogen Institute, Icahn School of Medicine at Mount Sinai, New York, NY 10029, USA; 10 Laboratorio de Virología Molecular, Centro Ciencia & Vida, Fundación Ciencia & Vida, Santiago 8581151, Chile; 11 Facultad de Medicina y Ciencia, Universidad San Sebastián, Santiago 7510157, Chile

**Keywords:** actinovirus, agnathovirus, *Bunyavirales*, *Hantaviridae*, hantavirid, hantavirus, loanvirus, mobatvirus, orthohantavirus, thottimvirus

## Abstract

The official classification of newly discovered or long-known unassigned viruses by the International Committee on Taxonomy of Viruses (ICTV) requires the deposition of coding-complete or -near-complete virus genome sequences in GenBank to fulfill a requirement of the taxonomic proposal (TaxoProp) process. However, this requirement is fairly new; thus, genomic sequence information is fragmented or absent for many already-classified viruses. As a result, taxon-wide modern phylogenetic analyses are often challenging, if not impossible. This problem is particularly eminent among viruses with segmented genomes, such as bunyavirals, which were frequently classified solely based on single-segment sequence information. To solve this issue for one bunyaviral family, *Hantaviridae*, we call on the community to provide additional sequence information for incompletely sequenced classified viruses by mid-June 2023. Such sequence information may be sufficient to prevent their possible declassification during the ongoing efforts to establish a coherent, consistent, and evolution-based hantavirid taxonomy.

## 1. Introduction

In 2017, the International Committee on Taxonomy of Viruses (ICTV) reacted to the rapid increase in virus discovery via metagenomics and metatranscriptomics by permitting an official virus classification based only on genomic sequence information, as long as that information is coding-complete (i.e., covers all open reading frames) or -near-complete (i.e., lacks only very few terminal or internal nucleotides that are difficult to resolve) [1]. This decision was based on the realization that a true depiction of the virosphere [2] would be impossible if individually characterizing viruses in the laboratory continued to be required; in addition, genomic sequence information enables large-scale phylogenetic analyses and thereby the establishment of evolutionary relationships among viruses in the absence of replicating representatives [1]. However, this decision was prospective, i.e., it applied to the assembly and evaluation of novel taxonomic proposals (TaxoProps) for the classification of newly discovered or previously unclassified viruses into taxa. (For an overview of the taxonomic classification process, the difference between species and virus, and classification methodologies, see Simmonds et al., 2023 [3]) Viruses classified by the ICTV prior to the 2017 decision remained classified even with, in some cases, the complete absence of genomic sequence information. Consequently, many virus taxa are currently mosaics of classified viruses that were placed into the official taxonomy through disparate methodologies using divergent classification criteria. This situation is untenable because the very goal of the ICTV is to “categorize the multitude of known viruses into a single classification scheme that reflects their evolutionary relationships, i.e., their individual phylogenies” [3,4].

The establishment of phylogenies requires genomic sequence information. More importantly, high quality of a virus genome sequence (e.g., sequence read depth and population analysis), redundancy (availability of equally high-quality genome sequences from different isolates of the same virus), completeness of the virus genome, and, in case of viruses with segmented genomes, each individual genome sequence derived from a single isolate enable improved and possibly complementary phylogenetic analyses using different parts of the genomes and their expression products—thus increasing confidence in the resulting taxonomic structures.

The ICTV first and foremost looks to its Study Groups to continuously improve the taxonomy of, typically, family-rank taxa with the long-term vision of achieving the ICTV goal of an accurate depiction of evolutionary virus relationships. Thus, it is largely up to these Study Groups to decide on virus classification criteria (e.g., minimal information necessary for classification) and taxon demarcation criteria (e.g., methodologies and metrics to be used for species and genus demarcation within a family). Here we express the intent of the ICTV *Hantaviridae* Study Group to resolve the classification problems plaguing the family *Hantaviridae*, with a first step envisioned to be an overhaul of the family based on analyses only, including viruses associated with coding-complete/-near-complete genome sequence availability in GenBank. We call on the hantavirid community to determine and/or provide missing sequence information for currently classified hantavirids to prevent their potential declassification and to provide such sequence information for currently unclassified viruses so that they can be assigned to species. With the annual ICTV deadline for the submission of TaxoProps this year being likely at the beginning of July, this information is needed by mid-June 2023 and then annually thereafter.

## 2. Current (2022–2023) Taxonomy of the Bunyaviral Family *Hantaviridae*

The official hantavirid taxonomy began relatively soon after the description of Hantaan virus (HTNV) in South Korean striped field mice (*Apodemus agrarius* (Pallas, 1771)) in 1976 [5,6,7] and the subsequent isolation of HTNV in cell culture in 1981 [8]. In 1983, first calls were issued to create an official genus for HTNV and its relatives [9,10,11,12]. In 1987, the genus *Hantavirus* was officially accepted by the ICTV as part of the family *Bunyaviridae* [13,14]. The subsequent discovery of hundreds of novel viruses assignable to this family resulted in the promotion of the family to the order *Bunyavirales* in 2017 [15]. Along with this promotion, the genus *Hantavirus* was promoted to the monogeneric family *Hantaviridae*, and all other already-classified viruses were assigned to the genus *Orthohantavirus* [15]. In 2019, the ICTV *Hantaviridae* Study Group designated DivErsity pArtitioning by hieRarchical Clustering (DEmARC) as the method of choice for the classification of novel hantavirids [16]. Analyses led to the establishment of the hantavirid genera *Loanvirus*, *Mobatvirus*, and *Thottimvirus* for divergent viruses discovered in bats and eulipotyphlans [17] and the genera *Actinovirus*, *Agnathovirus*, and *Reptillovirus* for divergent viruses discovered in fish and reptiles [18]. The family was then subdivided into four subfamilies: *Acanthavirinae* (*Actinovirus*), *Agantavirinae* (*Agnathovirus*), *Mammantavirinae* (*Loanvirus*, *Mobatvirus*, *Orthohantavirus*, and *Thottimvirus*), and *Repantavirinae* (*Reptillovirus*) [18] (for a more detailed history of the taxonomy of family *Hantaviridae*, see Kuhn and Schmaljohn, 2023 [19]). The current (2022–2023) taxonomy of *Hantaviridae* [20] is outlined in Table 1.

## 3. Future (2024–) Taxonomy of the Bunyaviral Family *Hantaviridae*

The 2019 taxonomic reorganization of the family *Hantaviridae* via DEmARC was limited to hantavirids for which coding-complete genome sequence information for the small (S) and medium (M) segments was available; concatenated S + M sequences were used for multiple-sequence alignment to infer phylogeny, and pairwise evolutionary distance (PED) values were calculated using a maximum-likelihood approach with a Whelan and Goldman (WAG) substitution model. A PED cut-off value of 0.1 was used for species demarcation within *Hantaviridae* [16]. The analysis was limited to the sequences of the S and M segments to maintain the previous hantavirid classification, which was largely based on phenotypic characters and limited protein sequence similarities of individual viruses [21], as much as possible and, in particular, to prevent the declassification of “important” orthohantaviruses (i.e., human pathogens) for which there was no or only fragmented sequence information for the large (L) segment [16].

However, the absence of L-segment sequence information in hantavirid taxonomic analyses is problematic for several reasons. First, the entire taxonomy of the realm *Riboviria*, which includes negarnaviricot *Hantaviridae*, is based on a single “hallmark gene”. This hallmark gene is the open reading frame (ORF) encoding an RNA-directed RNA polymerase (RdRp) [22,23], which in the case of hantavirids is a part of the L protein, encoded by the L segment. Thus, the absence of RdRp sequence information prevents the classification of a virus into this realm and, ipso facto, also into any lower-ranked ribovirian taxon. Second, as the name implies, L is by far the longest protein encoded by hantavirids; generally speaking, the main S-segment ORF is 1–3 kb long; the M-segment ORF is 3.2–4.9 kb long; and the L-segment ORF is 6.8–12 kb long (judged by GenBank entries). Thus, an analysis of, for example, concatenated S + M sequences ignores a substantial percentage of a hantavirid’s genome sequence. Third, sequence variability is unevenly distributed among hantavirid segments; the M-segment sequence is the least conserved, whereas the L-segment sequence is the most conserved. Both extremes can be used to achieve disparate goals, such as species and sub-species classifications, which require sequence divergence, and family and subfamily cohesiveness, which requires relatively conserved sequences. Finally, increasing structural information suggests that the Gn/Gc polyprotein encoded by hantavirid and other bunyaviral M segments (at least those of certain nairovirids, phenuivirids, peribunyavirids, and tospovirids) share a common ancestor with the membrane fusion machinery of distantly related positive-strand RNA viruses: alphaviruses (*Martellivirales*: *Togaviridae*), rubella virus (*Hepelivirales*: *Matonaviridae*), and flaviviruses sensu stricto (*Amarillovirales*: *Flaviviridae*) [24]; hence, the M segments are likely independent acquisitions in bunyaviral genome evolution. In addition, an increasing number of negarnaviricots that are being discovered in fungi and invertebrates do not appear to have M segments. Thus, although all currently classified hantavirids have M segments, the reliance on M segments within concatenated S + M data may become insufficient for family-wide analyses and may be inadequate for order-wide analyses.

The ICTV *Hantaviridae* Study Group decided to reassess the entire family for the 2023–2024 taxonomic cycle and plans to submit a TaxoProp proposing a new family taxonomy by the 2023 submission deadline (beginning of July). While the approaches/methodologies for reanalysis remain under discussion, a decision was made to only assess viruses for which there is S + M + L coding-complete or near-complete sequence information and deem all other viruses unclassifiable a priori. This stringent criterion would, at a minimum, result in the abolishment of six orthohantavirus species (Table 1, red), the declassification (removal from established species) of an additional 11 orthohantaviruses (Table 1, orange), and the possible renaming of two species (Table 1, purple) if word stem links between species and member viruses are desired to be maintained.

**Table 1:** Scheme of the 2022 [20] and projected 2023 [25] ^%^ taxonomy of the bunyaviral family *Hantaviridae*.

Genus	Species Name (2022)	Projected Species Name (2023)	Virus Name (Abbreviation)	Coding-Complete/-Near-Complete Genome Sequence Available in GenBank? ^^^
**Subfamily *Acanthavirinae***				
*Actinovirus*	*Batfish actinovirus*	*Actinovirus halieutaeae*	Wēnlǐng minipizza batfish virus (WEMBV)	Yes
	*Goosefish actinovirus*	*Actinovirus lophii*	Wēnlǐng yellow goosefish virus (WEYGV)	Yes
	*Perch actinovirus*	*Actinovirus bernense*	Bern perch virus (BRPV)	Yes
	*Spikefish actinovirus*	*Actinovirus triacanthodis*	Wēnlǐng red spikefish virus (WERSV)	Yes
**Subfamily *Agantavirinae***				
*Agnathovirus*	*Hagfish agnathovirus*	*Agnathovirus eptatreti*	Wēnlǐng hagfish virus (WEHV)	Yes
**Subfamily *Mammantavirinae***				
*Loanvirus*	*Brno loanvirus*	*Loanvirus brunaense*	Brno virus (BRNV)	Yes
	*Longquan loanvirus*	*Loanvirus longquanense*	Lóngquán virus (LQUV)	Yes
*Mobatvirus*	*Laibin mobatvirus*	*Mobatvirus laibinense*	Láibīn virus (LAIV)	Yes
	*Lena mobatvirus*	*Mobatvirus lenaense*	Lena virus (LENV)	Yes
	*Nova mobatvirus*	*Mobatvirus novaense*	Nova virus (NVAV)	Yes
	*Quezon mobatvirus*	*Mobatvirus quezonense*	Quezon virus (QZNV)	Yes
	*Xuan Son mobatvirus*	*Mobatvirus xuansonense*	Xuân Sơn virus (XSV)	Yes
*Orthohantavirus*	*Andes orthohantavirus*	*Orthohantavirus andesense*	Andes virus (ANDV)	Yes
			Castelo dos Sonhos virus (CASV)	No
			Lechiguanas virus (LECV = LECHV)	No
			Orán virus (ORNV)	No
	*Asama orthohantavirus*	*Orthohantavirus asamaense*	Asama virus (ASAV)	Yes
	*Asikkala orthohantavirus*	*Orthohantavirus asikkalaense*	Asikkala virus (ASIV)	Yes
	*Bayou orthohantavirus*	*Orthohantavirus bayoui*	bayou virus (BAYV)	Yes
			Catacamas virus (CATV)	Yes
	*Black Creek Canal orthohantavirus*	*Orthohantavirus nigrorivense*	Black Creek Canal virus (BCCV)	Yes
	*Bowe orthohantavirus*	*Orthohantavirus boweense*	Bowé virus (BOWV)	Yes
	*Bruges orthohantavirus*	*Orthohantavirus brugesense*	Bruges virus (BRGV)	Yes
	*Cano Delgadito orthohantavirus*	*Orthohantavirus delgaditoense*	Caño Delgadito virus (CADV)	Yes
	*Cao Bang orthohantavirus*	*Orthohantavirus caobangense*	Cao Bằng virus (CBNV)	Yes
			Liánghé virus (LHEV)	No
	*Choclo orthohantavirus*	*Orthohantavirus chocloense*	Choclo virus (CHOV)	Yes
	*Dabieshan orthohantavirus*	*Orthohantavirus dabieshanense*	Dàbiéshān virus (DBSV)	No
	*Dobrava-Belgrade orthohantavirus*	*Orthohantavirus dobravaense*	Dobrava virus (DOBV)	Yes
			Kurkino virus (KURV)	Yes
			Saaremaa virus (SAAV)	No
			Sochi virus (SOCV)	Yes
	*El Moro Canyon orthohantavirus*	*Orthohantavirus moroense*	Carrizal virus (CARV)	Yes
			El Moro Canyon virus (ELMCV)	No
			Huitzilac virus (HUIV)	Yes
	*Fugong orthohantavirus*	*Orthohantavirus fugongense*	Fúgòng virus (FUGV)	Yes
	*Fusong orthohantavirus*	*Orthohantavirus fusongense*	F ǔ s ō ng virus (FUSV)	No
	*Hantaan orthohantavirus*	*Orthohantavirus hantanense*	Amur virus (AMRV)	Yes
			Hantaan virus (HTNV)	Yes
			Soochong virus (SOOV)	Yes
	*Jeju orthohantavirus*	*Orthohantavirus jejuense*	Jeju virus (JJUV)	Yes
	*Kenkeme orthohantavirus*	*Orthohantavirus kenkemeense*	Kenkeme virus (KKMV)	Yes
	*Khabarovsk orthohantavirus*	*Orthohantavirus khabarovskense*	Khabarovsk virus (KHAV)	Yes
			Topografov virus (TOPV)	Yes
	*Laguna Negra orthohantavirus*	*Orthohantavirus negraense*	Laguna Negra virus (LANV)	No
			Maripa virus (MARV)	Yes
			Rio Mamoré virus (RIOMV)	Yes
	*Luxi orthohantavirus*	*Orthohantavirus luxiense*	Lúxī virus (LUXV)	Yes
	*Maporal orthohantavirus*	*Orthohantavirus maporalense*	Maporal virus (MAPV)	Yes
	*Montano orthohantavirus*	*Orthohantavirus montanoense*	Montaño virus (MTNV)	Yes
	*Necocli orthohantavirus*	*Orthohantavirus necocliense*	Necoclí virus (NECV)	No
	*Oxbow orthohantavirus*	*Orthohantavirus oxbowense*	Oxbow virus (OXBV)	No
	*Prospect Hill orthohantavirus*	*Orthohantavirus prospectense*	Prospect Hill virus (PHV)	Yes
	*Puumala orthohantavirus*	*Orthohantavirus puumalaense*	Hokkaido virus (HOKV)	Yes
			Muju virus (MUJV)	Yes
			Puumala virus (PUUV)	Yes
	*Robina orthohantavirus*	*Orthohantavirus robinaense*	Robina virus (ROBV) *	Yes
	*Rockport orthohantavirus*	*Orthohantavirus rockportense*	Rockport virus (RKPV)	Yes
	*Sangassou orthohantavirus*	*Orthohantavirus sangassouense*	Sangassou virus (SANGV)	Yes
	*Seewis orthohantavirus*	*Orthohantavirus seewisense*	Seewis virus (SWSV)	No
	*Seoul orthohantavirus*	*Orthohantavirus seoulense*	gōu virus (GOUV)	No
			Seoul virus (SEOV)	Yes
	*Sin Nombre orthohantavirus*	*Orthohantavirus sinnombreense*	New York virus (NYV)	No
			Sin Nombre virus (SNV)	Yes
	*Tatenale orthohantavirus*	*Orthohantavirus tatenalense*	Tatenale virus (TATV)	Yes
	*Thailand orthohantavirus*	*Orthohantavirus thailandense*	Anjozorobe virus (ANJZV)	Yes
			Serang virus (SERV)	No
			Thailand virus (THAIV)	Yes
	*Tigray orthohantavirus*	*Orthohantavirus tigrayense*	Tigray virus (TIGV)	Yes
	*Tula orthohantavirus*	*Orthohantavirus tulaense*	Adler virus (ADLV)	No
			Tula virus (TULV)	Yes
	*Yakeshi orthohantavirus*	*Orthohantavirus yakeshiense*	Yákèshí virus (YKSV)	No
*Thottimvirus*	*Imjin thottimvirus*	*Thottimvirus imjinense*	Imjin virus (MJNV)	Yes
	*Thottapalayam thottimvirus*	*Thottimvirus thottapalayamense*	Thottapalayam virus (TPMV)	Yes
**Subfamily *Repantavirinae***				
*Reptillovirus*	*Gecko reptillovirus*	*Reptillovirus hemidactyli*	Hǎinán oriental leaf-toed gecko virus (HOLGV)	Yes

Per the ICTV, viruses are real objects that are assigned to concepts/categories called taxa. Species, genera, subfamilies, families, and orders are taxa. Taxon names are always italicized and always begin with a capital letter. Virus names are not italicized and are not capitalized, except if the name or a name component is a proper noun [3,26]. This table lists the virus names with their correct (lack of) capitalization; ^%^ if ratified in the March 2023 ICTV-wide vote; ^^^ as judged by preliminary analyses of GenBank-deposited sequences but requiring careful reanalysis; * Robina virus might be a mobatvirus, possibly requiring reclassification [27].

On the other hand, the forthcoming analysis may include previously unclassified potential hantavirids for which sufficient S + M + L sequences have been deposited. A cursory survey revealed that at least two potential loanviruses, one potential mobatvirus, 16 potential orthohantaviruses, and one potential thottimvirus could be classified or be identified as isolates of already-classified viruses (Table 2, green).

## 4. Discussion

Hantavirid taxonomy is clearly in disarray, as exemplified by the numerous viruses with different names in the literature that may only represent isolates of other named viruses rather than being distinct viruses (species with several members in Table 1; numerous viruses listed in Table 2). Table 1 and Table 2 clarify that the diversity of hantavirids is only incompletely represented by the current taxonomy and family-wide analyses of hantavirids, and, therefore, the most appropriate sub-family taxon distribution is largely impossible because of the lack of evolutionary meaningful taxonomic markers (e.g., segment sequences, hallmark genes, and gene motifs). Even a relatively limited hantavirid classification inclusion criterion, such as the requirement of coding-complete/-near-complete sequences, will have a noticeable impact on the current taxonomy through the declassification of at least 17 orthohantaviruses (Table 1) and the classification of up to 20 hantavirids (Table 2). Taxonomic changes would likely be even more drastic if classification inclusion criteria were set more stringently; for instance, the ICTV *Hantaviridae* Study Group might additionally require that the S, M, and L genome segment sequences of a particular virus must be derived from the same isolate (rather than being a mosaic from isolates collected in different places at different times) and/or that specific sequencing standards [93,94] would have to be fulfilled to increase “trust” that the sequence is correct.

We call on the hantavirid and wider bunyaviral community to provide additional and/or improved sequence information for any incompletely sequenced putative hantavirid prior to mid-June 2023 to support the ICTV *Hantaviridae* Study Group’s current effort to establish an updated, coherent, consistent, and evolution-based hantavirid taxonomy. These sequences ought to be deposited into GenBank, ideally along with notifying the Study Group that additional information has become available for inclusion in analyses.

During the upcoming months, the ICTV *Hantaviridae* Study Group will make initial decisions on:

- the minimal requirement(s) for hantavirid classification (e.g., definitions of “near-complete genome sequence” and minimal sequence quality);
- the method(s) for hantavirid classification (e.g., DEmARC and/or pairwise sequence comparison [PASC]);
- the minimum input information (e.g., concatenated S + M or S + L or M + L or S + M + L genomic segment sequences; individual phylogenies and pairwise sequence comparisons for each genome segment);
- the possible resolution of “species complexes” (i.e., species that currently harbor more than one member virus [e.g., *Andes orthohantavirus*/*Orthohantavirus andesense*]);
- which particular sequences should be regarded as type/reference sequences for each species and be ultimately represented in The National Center for Biotechnology Information (NCBI) Reference Sequence (RefSeq) database.

All of these decisions will crucially depend on the availability of expanded high-quality hantavirid genomic sequence information. In the absence of this information, a decision may be forced to drastically reboot and simplify hantavirid taxonomy by removing the “virus status” from many unclassified hantavirids to discourage the use of their currently assigned names—effectively putting many hantavirids “on hold” until sufficient sequence information becomes available to assess their taxonomic statuses.

## Figures and Tables

**Table 2:** Unclassified potential hantavirids.

Potential Genus Affiliation ^^^	Virus ^%^	Coding-Complete/-Near-Complete Genome Sequence Available in GenBank? ^^^
*Actinovirus*	Murray-Darling rainbowfish hantavirus [28]	No
	pygmy goby hantavirus [29]	No
*Loanvirus*	Huángpí virus [30]	No
	Magboi virus [31]	No
	Méliandou hantavirus [32]	No
	Mouyassué virus [33]	Yes
	Ponan loanvirus [Not associated with a publication]	Yes
*Mobatvirus*	Đakrông virus [34]	Yes
	Kiwira virus [27]	No
	Altai virus [35,36]	No
	Makokou virus [37]	No
	Sarawak mobatvirus [38]	No
*Orthohantavirus*	Academ virus [39]	No
	Alto Paraguay virus [40]	No
	Amga virus [41]	No
	Anajatuba virus [42]	No
	Ape Aimé–Itapúa virus [43] ^1^	No
	Araraquara virus [44] ^1^	No
	Araucaria virus [45] ^1^	No
	Artybash virus [46] ^2^	Yes
	Ash River virus [47]	No
	Asturias virus [Not associated with a publication]	No
	Azagny virus [48]	No
	Belgrade virus [49]	No
	Bermejo virus [50] ^1^	No
	Biya river virus [Unpublished]	No
	Bloodland Lake virus [Not associated with a publication] ^3^	No
	Blue River virus [51] ^3^	No
	Boginia virus [52]	No
	Buenos Aires virus [53] ^1^	No
	Calabazo virus [54]	No
	Cajuru virus [55] ^1^	No
	Camp Riley virus [56]	No
	Central Plata virus [57] ^1^	No
	CGRn9415 virus [58] ^4^	No
	Dode virus [59]	No
	Fox Creek virus [Listed in [21]]	No
	Girard Point virus [60] ^4^	No
	hantavirus sp. strain Tamarin/BRA/SM22/2014 [Not associated with a publication] ^1^	No
	HoJo virus [61] ^5^	No
	Hu39694 virus [62] ^1^	No
	Iamonia virus [Listed in [21]]	No
	Isla Vista virus [63]	No
	Itapúa virus [64] ^1^	No
	Jaborá virus [43] ^1^	No
	Jemez Springs virus [47]	No
	jerboa hantavirus [65]	No
	Jīngmén Rattus norvegicus orthohantavirus 1 [Not associated with a publication]	Yes
	Juquitiba virus [66] ^1^	No
	Jurong virus [67] ^6^	No
	Kielder hantavirus [68]	No
	Landiras virus [69]	No
	Lanka virus [70]	Yes
	Leakey virus [71]	No
	Limestone Canyon virus [72]	No
	Lohja virus [73] ^2^	No
	Maciel virus [62] ^1^	No
	Malacky virus [74] ^7^	No
	Monongahela virus [75] ^3^	Yes
	Muleshoe virus [76]	No
	Ñeembucu virus [64] ^1^	No
	Paranoá virus [77] ^1^	No
	Pergamino virus [62] ^1^	No
	Playa de Oro virus [78]	No
	Powell Butte virus [Listed in [21]]	No
	prairie vole virus [Unpublished]	No
	Qiān Hú Shān virus/Qiāndǎo Lake virus [79]	No
	Rio Mearim virus [42]	No
	Río Segundo virus [80]	No
	Rusne virus [81]	Yes
	Sapporo rat virus [82] ^4^	No
	Sarufutsu virus [Not associated with a publication]	No
	Shěnyáng virus [83]	No
	Taimyr virus [84]	No
	Tanganya virus [85]	No
	Traemmersee virus [86] ^8^	Yes
	Tualatin River virus [Listed in [21]]	No
	Tunari virus [87] ^1^	No
	Uurainen virus [Not associated with a publication] ^2^	No
	Ussuri virus [Not associated with a publication] ^9^	Yes
	Vladivostok virus [Not associated with a publication]	No
	Wēnzhōu Niviventer niviventer orthohantavirus 1 [Not associated with a publication]	Yes
	Wùfēng Chodsigoa smithii orthohantavirus 1 [Not associated with a publication]	Yes
	Xìnyì virus [88] ^10^	Yes
	Yuánjiāng virus [83]	Yes
*Thottimvirus*	Dàhónggōu creek virus [89]	No
	Kilimanjaro virus [90]	No
	Uluguru virus [90]	No
	Wēnzhōu Suncus murinus thottimvirus 1 [Not associated with a publication]	Yes
*Reptillovirus*	skink hantavirus [91]	No
New?	coleopteran hanta-related virus OKIAV221 [92]	No
	plecopteran hanta-related virus OKIAV215 [92]	No

^^^ As judged by preliminary analyses of GenBank-deposited sequences but requiring careful reanalysis; ^%^ this list is based on a cursory analysis of the hantavirid literature and GenBank; the list may not be all-inclusive and may contain errors; ^1^ likely members of the species *Andes orthohantavirus*/*Orthohantavirus andesense* and possibly isolates of Andes virus (ANDV); ^2^ likely members of the species *Seewis orthohantavirus*/*Orthohantavirus seewisense* and possibly isolates of Seewis virus (SWSV); ^3^ likely members of the species *Sin Nombre orthohantavirus*/*Orthohantavirus sinnombreense* and possibly isolates of Sin Nombre virus (SNV); ^4^ likely members of the species *Seoul orthohantavirus*/*Orthohantavirus seoulense* and possibly isolates of Seoul virus (SEOV); ^5^ likely a member of the species *Hantaan orthohantavirus*/*Orthohantavirus hantanense* and possibly an isolate of Hantaan virus (HTNV); ^6^ likely a member of species *Thailand orthohantavirus*/*Orthohantavirus thailandense*, and possibly an isolate of Thailand virus (THAIV); ^7^ likely a member of the species *Tula orthohantavirus*/*Orthohantavirus tulaense* and possibly an isolate of Tula virus (TULV); ^8^ likely a member of the species *Tatenale orthohantavirus*/*Orthohantavirus tatenalense* and possibly an isolate of Tatenale virus (TATV); ^9^ likely a member of the species *Puumala orthohantavirus*/*Orthohantavirus puumalaense* and possibly an isolate of Puumala virus (PUUV); ^10^ likely a member of the species *Cao Bang orthohantavirus*/*Orthohantavirus caobangense* and possibly an isolate of Cao Bằng virus (CBNV).

## Data Availability

Not applicable.
